# A Protest against Changes in Nomenclature

**Published:** 1884-01

**Authors:** 


### A Protest Against Changes in Nomenclature.

The editor of the *Medical Press and Circular* does not like some of the changes recently made in the nomenclature of disease. Referring to a case reported as "adenoid disease of the rectum," he asks: "Is anything more conveyed by the term adenoid than by cancer? Sarcoma is another term now much used, and frequently to denote malignant disease. No benefit appears to us arising from these changes. On the contrary, they seem to lead to greater confusion. If a new disease be discovered, by all means let it be named according to its histology. But cancer is not new, nor does the microscope enable us to say positively what is cancer and what is not. Some years since the cancer cell was spoken of constantly. Where is it now? Is it a large or small cell? Angular or round? Take up any volume of pathological transactions and read the reports of the microscopical examination of malignant diseases and do we find anything definite stated? On the contrary, is not each report different from the other? And in numerous instances, after reading the report, we are left in total ignorance as to whether the diseased mass is malignant or not. This is not as it ought to be, and seems to us to present a point worthy of being handled in a different aspect from what has yet been done."

